# Impact of Human Activities on Woody Vegetation in Gallery Forests in the Mandara Mountains (Far North, Cameroon)

**DOI:** 10.1155/2024/9198533

**Published:** 2024-10-12

**Authors:** Hinémé Fanday, Tchobsala

**Affiliations:** University of Maroua, Faculty of Science, Department of Biological Sciences, P.O. Box 814, Maroua, Cameroon

**Keywords:** anthropogenic index, Cameroon, gallery forests, Mandara Mountains, woody vegetation

## Abstract

This study was conducted in the Mandara Mountains in Cameroon and aimed to assess the effects of human activities on woody vegetation in gallery forests, based on floristic inventories and observations made by the government. Firstly, the inventories were carried out in 150 plots of 1000 m^2^ each, installed on the banks of watercourses following the band of plant formations. In each plot, woody species were counted and those showing at least one sign of degradation were noted. Secondly, the survey was conducted in 18 administrative structures made up of delegations (MINFOF, MINADER, MINEPDED, and MINEPIA) and town halls. One hundred woody species, grouped into 63 genera and 30 families, have been inventoried, in which 45 species showed at least one sign of damage caused by human being. The species most affected are *Anogeissus leiocarpus* (67 stems), *Azadirachta indica* (46 stems), *Diospyros mespiliformis* (43 stems), *Acacia albida* (42 stems), *Andira inermis* (30 stems), *Acacia sieberiana* (23 stems), *Khaya senegalensis* (19 stems), *Ficus sycomorus* (13 stems), and *Acacia polyacantha* (10 stems). The most recurrent activity in the gallery forests is pruning (212 stems), followed by cutting (93 stumps), then picking (71 individuals). However, there are fewer debarked trees (11) and trees with fire trail (6). According to the responses provided, logging (77.78%), agriculture (72.22%), population growth (44.44%), grazing (33.33%), and bush fires (33.33%) are the main causes of the degradation of plant formations in the Mandara Mountains. These main factors could have a negative impact on biodiversity if appropriate integrated management measures are not taken. To maintain these vital ecosystems, an integrated management plan must be put in place, limiting human activities to a minimum.

## 1. Introduction

Forest strips with dense and exuberant vegetation, 20 to one hundred metres wide along the banks of rivers are known as gallery forests [1]. In tropical Africa, gallery forests are part of closed forest formations according to Yangambi's classification [2]. According to the definition in the biology dictionary, “gallery forest is a long narrow forest along the banks of a watercourse.” As complex ecological systems located at the land-water ecotone, riparian forests maintain generally high levels of beta and gamma diversity, exhibit high rates of nutrient cycling and productivity, provide specialized ecological functions, and exert strong influences on adjacent ecological systems [3]. These forests were important not only for the preservation of biological diversity, genetic diversity and network of biological interactions, but also related to the provision of ecosystem services, relative to land use, maintenance of water springs and ecological corridors, and aiming at the conservation of gene flow [4]. The benefits of forest can be seen from its contribution to the public wealth and as a source of livelihood to forest-dependent communities. Forests provide environmental sustainability in the form of carbon storage, habitat to several species, biodiversity, and storm mitigation [5]. At the same time, these gallery forests and their adjacent ecosystems are increasingly subject to disturbance by local residents, who derive a substantial proportion of their income from them. Uncontrolled fires disrupt the ecology and functionality of ecosystems, sometimes leading to their erosion [6]. They hinder the natural regeneration of woody species by destroying newly established individuals and shoots every year [7]. Over the last few decades, gallery forests have been threatened with extinction because of agricultural plots, fuelwood extraction, and other human pressures, despite their protection under Cameroon's Forestry Code. This explains the denudation and filling in of several watercourses in the Sudano-Sahelian zone of Cameroon. However, even if some of these gallery forests are quite degraded, there are still relics that are less anthropised in places. Despite the work carried out by several authors in the Mandara Mountains, notably Sandjong [8] on the phytoecological study of the Mozogo-Gokoro National Park: implications for sustainable management; Ibrahima [9] on the socioeconomic and environmental impact of the exploitation of Minawao vegetation; Kodji, Tchobsala, and Adamou [10] on the impact of refugees on the specific contribution and carbon stock of herbaceous plants in the Minawao savannah, there are few specific studies aimed at explaining the effects of human activities on gallery forests in this zone. Hence, the interest of this study is on the impact of anthropogenic activities on woody vegetation in gallery forests. The worsening state of gallery forest disturbance and the lack of participatory management drew our attention to this subject. In addition, the increasing threat to these resources from human activities leads us to fear that, in the near future, they may lose their ecological value or even disappear altogether. The problem that arises is that of conserving gallery forests. This raises the question of how to explain the impact of human activities on gallery forests? To answer this question, the aim is to assess the effects of human activities on gallery forests, based on floristic inventories and observations made by the Cameroon government, in order to propose sustainable management methods.

## 2. Materials and Methods

### 2.1. Study Site

**Figure 1:**
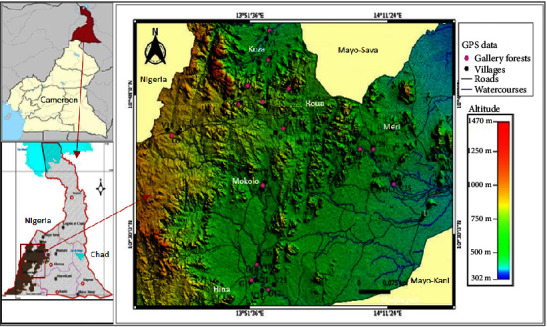
Location map of the study area.

The Mandara Mountains are located in the Far North Region of Cameroon, between latitudes 9°50′ and 11°35′ N, and between longitudes 13° and 14°E [11]. This area is subject to a dry tropical climate of the Sudano-Sahelian type. It is characterized by the alternation of two distinct seasons: a rainy season from May to September, and a rigorous dry season from October to April [12]. The average annual rainfall is about 800 mm. The annual average rainfall was about 800 mm in the Northern part and 1100 mm in the Southern part [13, 14]. The temperatures oscillated between 13°C (January) and 38°C (April); the annual average temperature was 28°C. The region's soils are highly diversified: sandy-clay and sandy soils on the hillsides and at the foot of the hills, strewn with stones of various sizes; humus-rich clay soils on the edges of the mayo; alluvial soils with a sandy-silty texture found on the plains and especially on the banks of watercourses; little-developed soils on the mountains; alluvial soils in the lowlands; and barren soils on the plains. The Mandara Mountains form a group of mountain massifs with an average altitude of 900 m. This is more visible on the location map where the altitude varies between 302 m (low altitude) and 1470 m (high altitude). The high-altitude zone lies along the border with the Republic of Nigeria, and the low-lying land at low altitude sweeps across the eastern part of the region. The Mandara Mountains are watered by several rivers that have their source in the mountains (Figure 1). These include the Mayo-Tsanaga which widens as it flows towards Maroua, the Mayo-Louti which widens as it flows towards Guider; the Mayo-Moskota which rises at the top of the Mandara Mountains, and the Mayo Mawa and Roua. These rivers dry up a few months after the rainy season [15]. The vegetation cover is made up of a combination of natural stands of endogenous forest species and planted species. The Sudano vegetation also varies, from the savannah, with its thorny steppes, to the mountains, where the same types of trees can be found but more stunted [16]. This vegetation has been severely degraded under the pressure of human activities (cutting of firewood, charcoal, service wood, bush fires, and overgrazing) and harsh climatic conditions [17]. The economic activities of the zone were based on extensive agriculture subsistence, extensive breeding, exploitation of resources, craft industry, and the small trade generating a substantial income for the poor rural families [14].

### 2.2. Data Collection

Data were collected in two stages: the floristic inventory phase and the survey phase.

#### 2.2.1. Floristic Inventory

**Figure 2:**
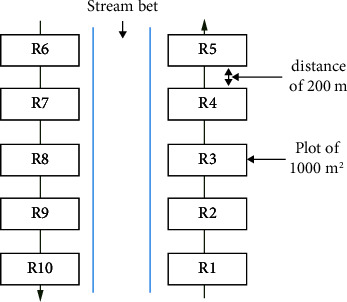
Phytosociological survey method.

The inventory was carried out in the 15 gallery forests selected in five districts (Koza, Roua, Mokolo, Hina, and Meri). In each arrondissement, gallery forests at least 1 km long were selected, and in each gallery forest, 10 1000 m^2^ plots (*R*_1_, *R*_2_,… *R*_10_) were set up on either side of the main watercourse on both banks. The plots were laid out perpendicular to the watercourse along the strips of vegetation at the edges of the watercourse. The distance between consecutive plots was 200 m (Figure 2). The phytosociological survey was carried out according to the quadrat method used by Gordon et al. [18]; Sambaré et al. [3]; Emmanuel, Tchobsala, and Adamou [19]. In each plot, all individuals with a circumference ≥ 5 cm were identified. This phase took place in autumn (September and October 2023), corresponding to the vegetation period, in order to facilitate species identification.

To determine anthropogenic index, standing dead individuals or individuals showing signs of human damage, i.e., cut, pruned, trimmed, barked, or burnt, were taken into account. In each plot, all trees with a circumference ≥ 5 cm were identified. Individuals showing signs of human damage, such as cut, pruned, or trimmed trees, scratches, traces of fire, and dead trees, were noted and counted by species on site; this enabled us to calculate the anthropogenic index.

#### 2.2.2. Survey

To assess the impact of human activities on gallery forest woody species in the Mandara Mountains, a survey was carried out in the delegations (MINFOF, MINADER, MINEPDED, MINEPIA, and Town hall) located in the surveyed districts covered by the study area (Koza, Mokolo, Roua, Hina, and Meri). Thus, 18 administrative structures (delegations and Town halls) were interviewed, distributed as follows: MINFOF (4 structures or offices), MINADER (5), MINEPDED (one), MINEPIA (3), and Town hall (5). Based on their observations in the field, these delegations provided information on the impact of human activities on forest resources in general and gallery forests in particular. They also proposed a number of solutions for conserving gallery forests in this part of the country. Note that these selected delegations have a direct link with the environment (MINEPDED), forestry (MINFOF), agro-pastoral activities (MINADER and MINEPIA), and the Town hall, which is involved in all these sectors of activity.

### 2.3. Data Analysis

The raw data collected in the field was entered and classified in a database using Microsoft Excel 2016. XLSTAT 2014 was used to analyze the statistical data. Analysis of variance (ANOVA) was used to compare the means of the anthropogenic indices and principal component analysis (PCA) to study the correlation between these indices. The significance level taken into account for this test is alpha = 5%. For the survey data, the response rate or citation frequency was calculated as a percentage.

#### 2.3.1. Analysis of Inventory Data

The floristic data obtained from the vegetation survey were processed. The following parameters were calculated.

Density (*D*) is the number of individuals per unit area. It is expressed as the number of individuals/ha. It is obtained by dividing the total number of individuals in the sample by the surface area sampled.

(1) $$\begin{array}{c} D=\frac{n}{S}, \end{array}$$

where *n*: total number of individuals in the sample considered and *S*: sample area in ha [20].

The Shannon diversity index (*H*′) was calculated using the following formula:

(2) $$\begin{array}{c} H^{\prime }=-\overset{s}{\underset{i=1}{\sum }}\text{Pi }\ln\text{ Pi}, \end{array}$$

where *S* is the number of species, Pi is the proportion of individuals of the *i*th species expressed as a proportion of total cover in the sample, and ln: the natural logarithm [21]. Diversity is low when *H*′ < 3 bits, medium if 3 < H′ < 4 bits, and high when *H*′ > 4 bits.

The anthropogenic index (Id), expressed as a percentage, is equal to the ratio between the number of individuals of the species showing at least one damage linked to human activity (*N* damage) and the total number of individuals of the species (*N* adults + *N* regenerated + *N* dead) according to the formula:

(3) $$\begin{array}{c} \text{Id}=\frac{N\text{ damage}}{N\text{ adults}+N\text{ reg}.+N\text{ dead}}\times 100. \end{array}$$

Human-related damage is mainly damage due to cutting, pruning, picking, debarking, and fires [20].

#### 2.3.2. Analysis of Survey Data: Frequency of Quotation

The citation frequency (CF) is the ratio of the number of informants who cited a particular index of the degradation (*n*) divided by the total number of informants or interviewees. It calculated as follow:

(4) $$\begin{array}{c} \text{FC}=\frac{n}{N}\times 100. \end{array}$$

## 3. Results and Discussion

### 3.1. Floristic Composition and Anthropogenic Indices

#### 3.1.1. Floristic Composition and Diversity

A total of 100 woody species grouped into 67 genera and 30 families is recorded in the gallery forests of the Mandara Mountains (Table 1). This result is similar to that of Nascimento et al. [4], who recorded 108 species belonging to 41 families in the gallery forests flora in Brazilian Centrale Plateau. This similarity shows that our study area and that of the above-mentioned author have the same floristic characteristics. However, our result is much lower than that of Veneklaas et al. [22], who listed 147 species distributed in 45 families in the gallery forest types in a Colombian savannah landscape and that of Marimon et al. [23], who counted 137 species belonging to 50 families in a gallery forest at the Cerrado/Amazonia boundaries in Brazil. This can be explained by the fact that their study areas are more diverse than the Mandara Mountains. The same is true of Gordon et al. [18], who inventoried 124 species in 46 families in the gallery forests in the North West Cameroon. The same is true of the results of Sambaré et al. [3] in the gallery forests of the rivers and streams of the southern Sudan sector of Burkina Faso, where they identified 127 species grouped into 47 families and those of Armenteras et al. [24], who identified 128 species belonging to 35 families in the tropical gallery forests. However, it is much higher than that of Barbosa and Pizo [25], who identified only 84 species in 29 families in a planted gallery forest in Brazil. This difference may be due to several contrasting factors in the Sudano-Sahelian environment of Cameroon. Human activities in the gallery forests (logging, bush fires, agriculture, and grazing), rainfall deficits, high temperature, and altitudinal gradients could all have an impact on the regeneration and resilience of woody species. It should also be noted that the underlying factors (population growth and infrastructure development) that generate the need for land and wood have a direct or indirect impact on the diversity of flora in this zone.

The Shannon index for all arrondissements is 3.04 bits on average. The index is highest in Mokolo (3.22 bits) and lowest in Koza (2.86 bits). This result corroborates that of Kassa, Deribie, and Walle [26] according to which Shannon diversity varies between 2.26 and 3.72 bits in Gosh-Beret Dry Evergreen Forest Patch, Northeast Ethiopia.

The average density is 239 stems·ha^−1^. It is high in the commune of Mokolo with 266 stems·ha^−1^, but low in the commune of Meri with 220 stems·ha^−1^. The overall density obtained (239 stems·ha^−1^) is similar to that of Todou et al. [27] who obtained 239.17 stems·ha^−1^ in Nyé'été forest, south region of Cameroon.

#### 3.1.2. Anthropogenic Index

**Figure 3:**
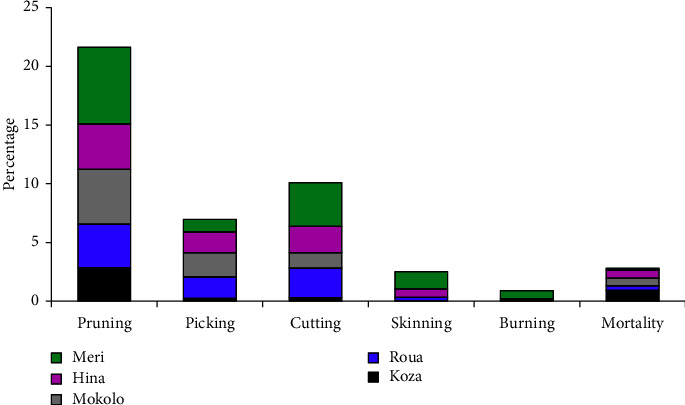
Anthropogenic index observed in gallery forests.

To assess the state of degradation of gallery forests in the Mandara Mountains, anthropogenic indexes are calculated. These indexes vary according to the types of activity practiced by the populations. The most noticeable index in gallery forests is pruning, with 4.32% on average, and the most noticeable is in the district of Meri with 6.52%. This activity is much more common among local people for the following reasons: firstly, it is one of the techniques used to harvest firewood and service wood so as not to destroy the tree. It is also a way of pruning the tree so that it can regenerate, unlike picking, which often disrupts regeneration. Pruning is used for forage species (*Celtis integrifolia, Stereospermum kunthianum, Adansonia digitata, Khaya senegalensis, Ficus sur, Leucaena leucocephala, Sterculia setigera,* and *Vitex doniana*). The least common activity in gallery forests is bushfire with 0.3%. This index is not observed in the Roua and Hina districts. In the Koza and Mokolo districts, the species do not show signs of degradation due to bark removal. However, the few species that have been barked are often used in traditional pharmacopoeia to treat illnesses. As for the mortality rate, a few individuals died on the ground in some places, with the rate varying from 0.13% in Meri to 0.91% in Koza. The average mortality rate was 0.56% (Figure 3).

**Figure 4:**
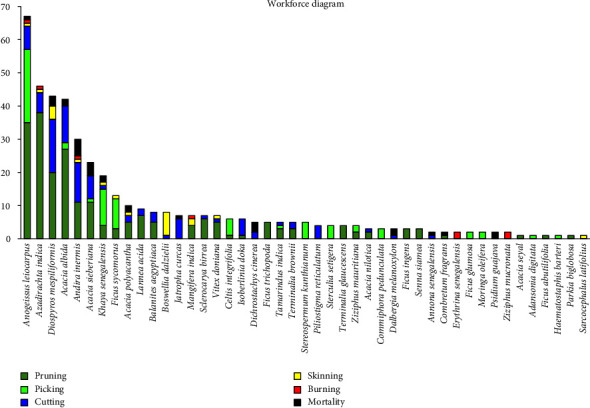
Species numbers according to anthropogenic indices.

Figure 4 shows the number of individuals of each species according to the damage caused by human activities. In all, 45 species identified in the gallery forests show at least one sign of human damage. The most important are *Anogeissus leiocarpus* (67 stems), *Azadirachta indica* (46 stems), *Diospyros mespiliformis* (43 stems), *Acacia albida* (42 stems), *Andira inermis* (30 stems), *Acacia sieberiana* (23 stems), *Khaya senegalensis* (19 stems), *Ficus sycomorus* (13 stems), and *Acacia polyacantha* (10 stems). The number of individuals of each of these species heavily affected is greater than or equal to 10. The recurrent activity in the gallery forests is branch pruning, which affects 28 species of which *Azadirachta indica* (38 stems), *Anogeissus leiocarpus* (35 stems), *Acacia albida* (27 stems), *Diospyros mespiliformis* (20 stems), *Andira inermis* (11 stems), and *Acacia sieberiana* (11 stems) are the most pruned. The cutting of trees is much more observed in *Diospyros mespiliformis* (16 stems), *Andira inermis* (12 stems), and *Acacia albida* (11 stems). The population to feed their livestock with fodder also practises picking. This pressure weighs much more heavily on species such as *Anogeissus leiocarpus* (22 stems), *Khaya senegalensis* (11 stems), and *Ficus sycomorus* (9 stems). However, there are fewer species showing signs of bushfire burns; apart from the eight individuals recorded. Kodji, Tchobsala, and Adamou [10] index species such as *Anogeissus leiocarpus*, *Azadirachta indica*, and *Acacia albida* as being the most exploited in the Minawao zone. A one-way analysis of variance gives *p* value ≤ 0.001. To this end, the null hypothesis (*H*_0_) can be rejected. Therefore, there is a statistically significant difference between the means of the anthropogenic indices at the significance level, *α* = 0.05.

**Figure 5:**
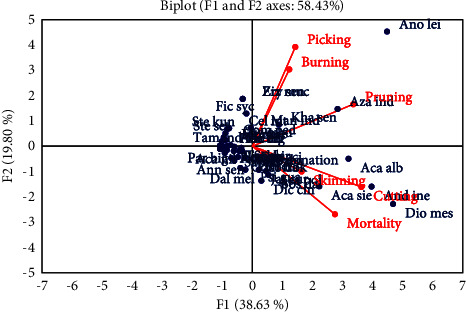
Principal component analysis between anthropogenic indices.

Principal component analysis (PCA) is made to study the relationships between these anthropogenic indices. It shows that 38.63% of information, while the second dimension explains 19.80%. These dimensions explain 58.43% of total information contained in the dataset. The correlation circle shows that cutting, pruning and mortality are positively correlated with F1, with their correlation coefficients *r* = 0.894^∗^, *r* = 0.826^∗^, and *r* = 0.677^∗^, respectively. While picking and burning are correlated with F2 with *r* = 0.692^∗^ and *r* = 0.536^∗^, respectively. However, skinning is not well represented in the factorial design and does not play a significant role in the dataset, but is correlated instead with other dimensions of the PCA. Between cutting vs pruning (*r* = 0.674^∗^), then cutting vs mortality (*r* = 0.657^∗^), a correlation is positive and statistically significant (^∗^). On the other hand, correlation is almost zero between picking vs mortality (*r* = 0.030), burning vs mortality (*r* = 0.032), skinning vs picking (*r* = 0.042), skinning vs burning (*r* = 0.048), and picking vs cutting (*r* = 0.078). Their correlation coefficients are not significantly different from zero. For the observations, species such as *Anogeissus leiocarpus*, *Diospyros mespiliformis*, *Andira inermis*, *Azadirachta indica*, and *Acacia albida* are scattered from the others (Figure 5). These species have suffered considerable damage from human activities. They are therefore the most threatened species in the gallery forests. On the other hand, those forming a dense cloud of points at the origin of the axes are less disturbed by human action.

### 3.2. Management of Gallery Forests in the Mandara Mountains

#### 3.2.1. Causes of Gallery Forest Degradation

Degradation of vegetation cover in general and gallery forests in particular often occurs because of ongoing human activities. The various factors causing this degradation are many and varied, as summarized in Table 2 below. This table shows that the main causes are logging, with a response rate of 77.78%, of which a town hall provided 27.78% of response rate; 22.22% came from MINFOF and MINADER and 5.56% from MINEPDED. Uncontrolled tree felling can leave a land bare if it is not reforested. Then, there are agriculture (72.22%), population growth (44.44%), bush fires, and grazing (33.33% each), which are equally distributed according to the opinions and knowledge of the leaders. Tchobsala et al. [28] made a same observation according to whom wood cutting (53.56%), agriculture (20.23%), and overgrazing (6.21%) are the main anthropogenic activities on park vegetation in North Cameroon. Sandjong [8] also noted in the Mozogo district that wood harvesting (51.11%), bushfires (44.44%), and grazing (31.11%) are the most common on the Mandara Mountains. It is also similar to the work of Mekideche, Brakchi-Ouakour, and Kadik [29], who demonstrated that recurrent fires and overgrazing are the main disturbances to forest formations present in northern Algeria. In addition, it should be noted that removal of bark and leaves disrupts the resilience and regeneration of vegetation. A tree that has been stripped of its bark cannot withstand bushfire or bad weather. The causes of tree mortality in the forests are often due to wildfire and bark removal. Abuse of the latter can undoubtedly lead to the death of a tree. According to the perception of the competent authorities in the field, the exploitation of nontimber forest products (11.11%) is a least significant factor affecting forest biodiversity. It is true that this factor has a negative impact on trees, but not in a direct way.

However, the consequences of these human factors can make species more vulnerable. The disappearance of biodiversity, reduced rainfall, global warming, soil erosion, and desertification are often the consequences of such phenomena in the Mandara Mountains in Cameroon. Although these effects are not visible in a short term, they may be noticeable in the long term. Global warming is due to increasing in the high level of greenhouse gases such are CO_2_, N_2_, H_2_S, CO, CH_4,_ CFC, and other pollutants likely to affect the ozone layer. Air pollution depends on the emission of these gases and the residual concentration of pollutants in the ambient air. It is mainly due to human activity, such as vehicle exhaust fumes, smoke from bush fires, and miscellaneous combustion. Water pollution is caused by the accumulation of chemicals (fertilizers, pesticides, and herbicides) used in agriculture. In addition, there are development of infrastructures, low agricultural yields, destruction of grazing land, problem of food security, pollution, and conquest of other forest environments. According to Letouzey [17], the human influence on vegetation can manifest itself in many ways: old or recent migrations, large or small, agricultural nomadism, establishment of various crops, development of roads and means of communication attracting or repelling villages, and expanding towns.

#### 3.2.2. Some Proposed Solutions for the Management of Gallery Forests in the Mandara Mountains

Drawing on the experience gained and results obtained in terms of knowledge of biodiversity in the Mandara Mountains, Cameroonian government is aware of the many significant advantages that gallery forests offer to a population. Aware of the risks associated with their degradation, it will therefore be necessary to put in place an integrated management strategy for a preservation of the environment as a whole and a conservation of gallery forests in particular. A number of solutions relating to human activities are proposed by the district delegations surveyed (Table 3). According to them, these include creating a grazing area, restoring and respecting a grazing management plan, and developing cattle tracks or grazing corridors. This will ensure good grazing management to limit transhumance and trampling of cattle in the gallery forests. As far as the rational and sustainable use of forest resources is concerned, wood cutting should be kept to a minimum, or at least a legal tax should be paid to allow controlled logging. Improved stoves and other energy sources should be used to limit the excessive consumption of firewood. With regard to cultivation practices on the banks of watercourses, it will be necessary to respect the boundaries between fields and gallery forests. Agroforestry and forestry are also alternatives for conserving forest species. Slash-and-burn agriculture around gallery forests should also be avoided. To manage the surrounding environment properly, we need to put an end to bush fires and raise awareness among the population about how to carry out their activities and about the importance of forests in their daily lives. This popularization of the results of investigations into conservation of gallery forests in the Mandara Mountains will present in the form of guidelines that can apply by players likely to be interacting with the resources or involve in their sustainable management. With a forest area of 19.5 million hectares excluding gallery forests, i.e. just under half the national territory, Cameroon's forestry sector makes a significant contribution to the national economy through the creation of jobs and sociocommunity infrastructure, and above all through the tax revenues generated [30].

**Figure 6:**
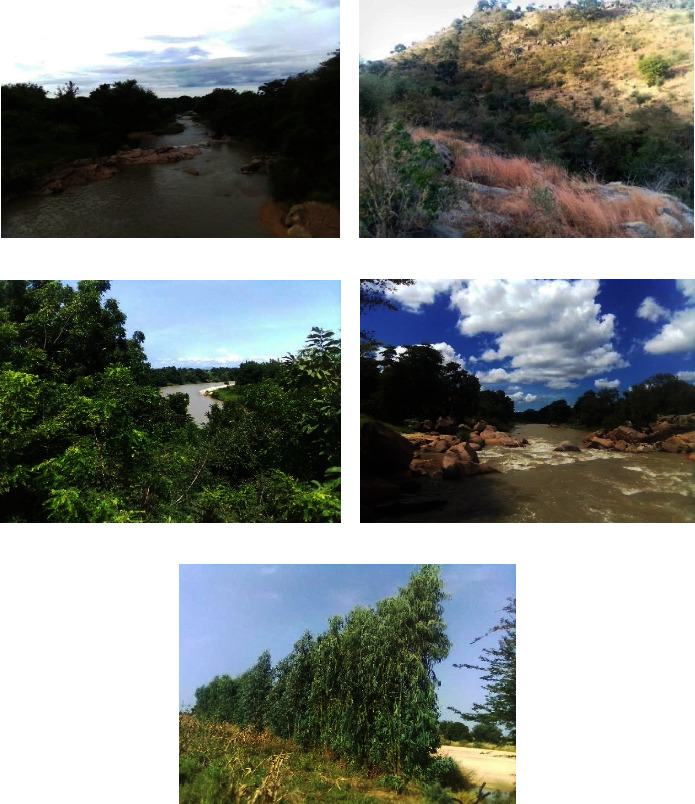
Gallery forests in the Mandara Mountains.

Figure 6 illustrates the physiognomy of some of gallery forests studied in the Mandara Mountains: case of the gallery forests in Mokolo (a), Koza (b), Hina (c), Roua (d), and Meri (e). They are made up of natural vegetation and planted species dominated by shrubs.

This research was conducted with a view to contributing to the management of forest resources as a whole. It can help the local, regional, national, and even international community, and political decision-makers to make decisions regarding environmental impact assessment. Nowadays, biodiversity conservation has become a real concern and is at the center of major environmental issues. The management of gallery forests today can help to solve the problems associated with climate change and contribute to the carbon stock. It can also help to combat bank erosion, maintain soil stability, and purify water. A well-managed gallery forest is an ecosystem with multiple roles. It is a place of refuge for other living creatures, such as wildlife, especially in areas where forest cover is almost nonexistent.

However, as an open formation, gallery forests sometimes have their limits when it comes to managing this ecosystem in situ. The lack of means and sophisticated tools to delimit the perimeter of these formations, on the one hand, and noncompliance with the law and forestry regulations, on the other, can become complex. Further studies would be useful to assess the potential of gallery forests.

## 4. Conclusion

At the end of this research project, the aim was to assess the impact of human activities on gallery forests in the Mandara Mountains. Based on inventory data, we found that these forests are moderately rich in diversity, with an even distribution of individuals per hectare, despite the pressure of human activity that is already perceptible through the anthropogenic indices observed in the field. Based on information gathered from the relevant administrative authorities, activities such as logging and extensive farming are the main causes of the degradation of the vegetation cover in the Mandara Mountains. To this end, the conservation of the biological resources of the gallery forests and their periphery is of vital importance for their rich biodiversity and productivity. Changes are likely in the long term if nothing is done to maintain the balance of these plant formations. To this end, practical measures need to be taken, such as setting up research, ecological monitoring and evaluation strategies, and even planning and applying the principles of forest resource conservation while limiting the negative impact of man on all gallery forests.

## Figures and Tables

**Table 1:** Composition and floristic diversity of woody species in gallery forests.

Districts	Species	Genera	Family	Shannon index (bits)	Density (stems·ha^−1^)
Koza	62	45	24	2.86	244
Roua	49	34	17	3.11	228
Mokolo	63	45	25	3.22	266
Hina	53	32	18	2.95	237
Meri	51	38	21	3.06	220
Total	100	63	30	3.04	239

**Table 2:** Main factors of the degradation of gallery forests in the Mandara Mountains.

Causes	MINFOF	MINADER	MINEPDED	MINEPIA	Town hall	Total
Wood cutting	22.22	22.22	5.56	—	27.78	77.78
Agriculture	16.67	11.11	5.56	11.11	27.78	72.22
Bush fires	5.56	5.56	5.56	—	16.67	33.33
Population growth	16.67	5.56	5.56	11.11	5.56	44.44
Grazing	5.56	5.56	5.56	11.11	5.56	33.33
NTFP harvesting	5.56	—	—	—	5.56	11.11

**Table 3:** Solutions proposed by the Cameroon government to manage gallery forests.

Proposed solutions	MINFOF	MINADER	MINEPDED	MINEPIA	Town hall	Total
Limit abusive wood cutting	16.67	11.11	—	—	27.78	55.56
Pay a tax for legal exploitation	16.67	5.56	—	5.56	16.67	44.44
Prune trees or branches	11.11	5.56	5.56	—	—	22.22
Reforestation	5.56	5.56	5.56	—	5.56	22.22
Create private forests	5.56	—	—	—	—	5.56
Use other energy sources	—	5.56	5.56	—	—	11.11
Use improved fireplaces	—	5.56	5.56	—	—	11.11
Prohibit cultivation in gallery forests	11.11	11.11	—	5.56	16.67	44.44
Raise awareness of the importance of trees	—	22.22	5.56	—	—	27.78
Practicing agroforestry	11.11	—	—	—	—	11.11
Practicing forestry	—	—	5.56	—	—	5.56
Respect the limits of field and gallery	5.56	5.56	—	—	5.56	16.67
Avoid slash and burn farming	—	5.56	—	5.56	22.22	33.33
Create a grazing/ranching area	11.11	11.11	—	5.56	22.22	50.00
Respect the grazing management plan	—	—	5.56	5.56	—	11.11
Restore grazing	—	—	5.56	5.56	—	11.11
Developing grazing corridors	5.56	—	—	5.56	—	11.11
Plant forage species	—	—	5.56	—	—	5.56
Raise in the enclosure	5.56	—	—	11.11	5.56	22.22
Ban transhumance	5.56	—	—	—	—	5.56
Ban bush fires	5.56	11.11	—	11.11	16.67	44.44
Avoid late bush fires	5.56	—	—	—	—	5.56
Controlling early fires	5.56	—	—	—	—	5.56
Educate the public and raise awareness	5.56	5.56	5.56	5.56	—	22.22

## Data Availability

The data that support the findings of this study are included within the article.
